# Supplementary material for: Toxin expression in snake venom evolves rapidly with constant shifts in evolutionary rates
Source: Proc Biol Sci. 2020 Apr 29;287(1926):20200613. doi: 10.1098/rspb.2020.0613 (PMC7282918; doi:10.1098/rspb.2020.0613)
Supplement: Phylogeny and relative abundance of 10 toxin families from venom transcriptome of 52 species of venomous snake. [file rspb20200613supp1.pdf]

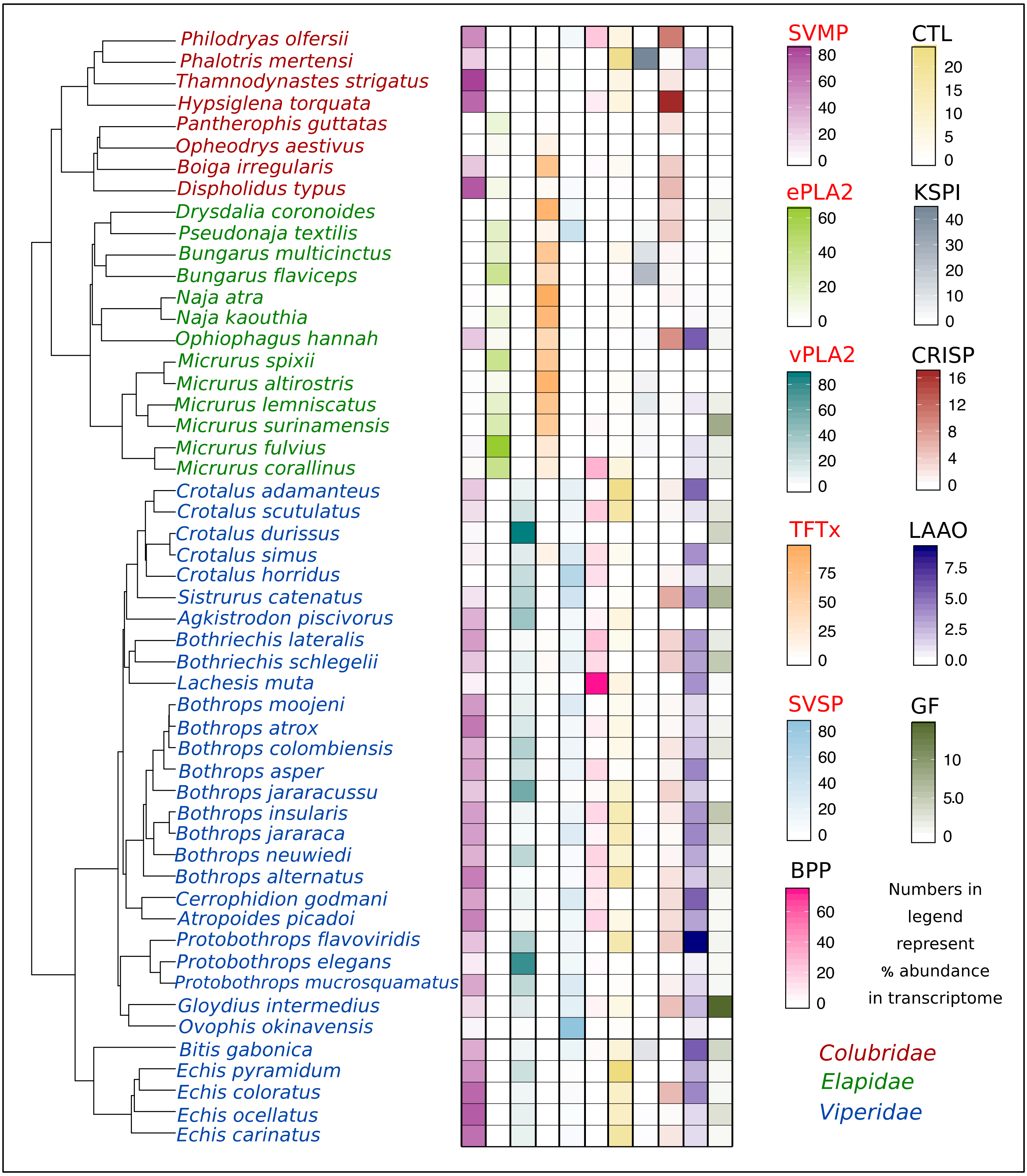

Philodryas olfersii
Phalotris mertensi
Thamnodynastes strigatus
Hypsiglena torquata
Pantherophis guttatas
Opheodrys aestivus
Boiga irregularis
Dispholidus typus
Drysdalia coronoides
Pseudonaja textilis
Bungarus multicinctus
Bungarus flaviceps
Naja atra
Naja kaouthia
Ophiophagus hannah
Micrurus spixii
Micrurus altirostris
Micrurus lemniscatus
Micrurus surinamensis
Micrurus fulvius
Micrurus corallinus
Crotalus adamanteus
Crotalus scutulatus
Crotalus durissus
Crotalus simus
Crotalus horridus
Sistrurus catenatus
Agkistrodon piscivorus
Bothriechis lateralis
Bothriechis schlegelii
Lachesis muta
Bothrops moojeni
Bothrops atrox
Bothrops colombiensis
Bothrops asper
Bothrops jararacussu
Bothrops insularis
Bothrops jararaca
Bothrops neuwiedi
Bothrops alternatus
Cerrophidion godmani
Atropoides picadoi
Protobothrops flavoviridis
Protobothrops elegans
Protobothrops mucrosquamatus
Gloydius intermedius
Ovophis okinavensis
Bitis gabonica
Echis pyramidum
Echis coloratus
Echis ocellatus
Echis carinatus
SVMP 80 60 40 20 0
CTL 20 15 10 5 0
ePLA2 60 40 20 0
KSPI 40 30 20 10 0
vPLA2 80 60 40 20 0
CRISP 16 12 8 4 0
TFTx 75 50 25 0
LAAO 7.5 5.0 2.5 0.0
SVSP 80 60 40 20 0
GF 10 5.0 0
BPP 60 40 20 0
Numbers in legend represent % abundance in transcriptome
Colubridae
Elapidae
Viperidae

Fig. 1: Phylogeny (51) **and relative abundance of 10 toxin families from venom transcriptome of 52 species of venomous snake. Snakes use different levels of each toxin family in their venom with a clear preference between families. The toxin names in red denote the most abundant and widespread toxin families in venomous snakes. Except for the PLA2s, which have distinct lineages in colubrid and elapid vs. viperid snakes, all toxins seem to present at some level each of the three families of venomous snakes. SVMP: Snake Venom Metalloprotease, SVSP: Snake Venom Serine Protease, ePLA2: Phospholipase A2 (I), vPLA2: Phospholipase A2 (II), TFTx: Three Finger Toxin, CRISP: Cysteine-Rich Secretory Protein, CTL: C-type Lectin, LAAO: L-amino acid oxidase, KSPI: Kunitz-type serine protease inhibitors, BPP: Bradykinin-potentiating peptide, GF: Growth factors.**
