# Supplementary material for: Toxin expression in snake venom evolves rapidly with constant shifts in evolutionary rates
Source: Proc Biol Sci. 2020 Apr 29;287(1926):20200613. doi: 10.1098/rspb.2020.0613 (PMC7282918; doi:10.1098/rspb.2020.0613)
Supplement: Rapid pulsed evolution modelled as a Lévy process explain toxin expression evolution in snake venom better than conventional BM, OU, and EB models. [file rspb20200613supp2.docx]

| Toxin family | BM | OU | EB | Pulsed |
| --- | --- | --- | --- | --- |
| *BPP* | 0.039 | 0.014 | 0.051 | 0.377 |
| *CRISP* | 0.031 | 0.011 | 0.011 | 0.455 |
| *CTL* | 0.002 | 0 | 0 | 0.979 |
| *GF* | 0.171 | 0.062 | 0.062 | 0.465 |
| *KSPI* | 0 | 0 | 0 | 0.711 |
| *LAAO* | 0.123 | 0.021 | 0.122 | 0.335 |
| *SVMP* | 0.002 | 0 | 0 | 0.678 |
| *SVSP* | 0.127 | 0 | 0.046 | 0.349 |
| *TFTx* | 0 | 0 | 0 | 0.976 |
| *vPLA2* | 0.089 | 0.033 | 0.032 | 0.363 |
| *ePLA2* | 0 | 0 | 0 | 0.900 |

**Table 1: Rapid pulsed evolution modelled as a Lévy process explain toxin expression evolution in snake venom better than conventional BM, OU, and EB models. Model fits (weighted AIC) for BM, OU, EB, and Pulsed model of phenotypic evolution computed in *pulsR*** (13)**. Red indicates best fit.** **We use the AIC weight to determine which model best suits our data. The values in our table represent AIC weights for each of the 9 models we tested (BM, OU, EB, and 6 pulsed models). In all cases the pulsed models were favoured as compared to the non-pulsed models. However, each pulsed model had very similar weights, which make it difficult to determine which pulsed model is better. For that reason, we club them together and report the highest AIC weight*.***
